# Pneumonia-Specific *Escherichia coli* with Distinct Phylogenetic and Virulence Profiles, France, 2012–2014

**DOI:** 10.3201/eid2504.180944

**Published:** 2019-04

**Authors:** Béatrice La Combe, Olivier Clermont, Jonathan Messika, Matthieu Eveillard, Achille Kouatchet, Sigismond Lasocki, Stéphane Corvec, Karim Lakhal, Typhaine Billard-Pomares, Romain Fernandes, Laurence Armand-Lefevre, Sandra Bourdon, Jean Reignier, Vincent Fihman, Nicolas de Prost, Julien Bador, Julien Goret, Frederic Wallet, Erick Denamur, Jean-Damien Ricard, on behalf of the COLOCOLI group

**Affiliations:** Infection, Antimicrobiens, Modélisation, Évolution, Paris, France (B. La Combe, O. Clermont, J. Messika, T. Billard-Pomares, R. Fernandes, L. Armand-Lefevre, E. Denamur, J.-D. Ricard);; Université Paris Diderot, Paris (B. La Combe, O. Clermont, J. Messika, T. Billard-Pomares, R. Fernandes, L. Armand-Lefevre, E. Denamur, J.-D. Ricard);; Hôpital Louis Mourier, Colombes, France (B. La Combe, J. Messika, T. Billard-Pomares, R. Fernandes, J.-D. Ricard);; Centre Hospitalier Universitaire, Angers, France (M. Eveillard, A. Kouatchet, S. Lasocki);; Centre Hospitalier Universitaire, Nantes, France (S. Corvec, J. Reignier);; Hôpital Laënnec, Nantes (K. Lakhal); Hôpital Bichat, AP-HP, Paris (L. Armand-Lefevre, E. Denamur);; Centre Hospitalier Départemental Vendée, La Roche-sur-Yon, France (S. Bourdon);; Hôpital Henri Mondor, AP-HP, Créteil, France (V. Fihman, N. de Prost);; Centre Hospitalier Universitaire Bocage Central, Dijon, France (J. Bador);; Centre Hospitalier Universitaire Pellegrin, Bordeaux, France (J. Goret);; Centre Hospitalier Régional Universitaire, Lille, France (F. Wallet)

**Keywords:** Nosocomial infection, ventilator-associated pneumonia, *Escherichia coli*, virulence factors, antimicrobial resistance, bacteria, respiratory infections, pneumonia, France

## Abstract

In a prospective, nationwide study in France of *Escherichia coli* responsible for pneumonia in patients receiving mechanical ventilation, we determined *E. coli* antimicrobial susceptibility, phylotype, O-type, and virulence factor gene content. We compared 260 isolates with those of 2 published collections containing commensal and bacteremia isolates. The preponderant phylogenetic group was B2 (59.6%), and the predominant sequence type complex (STc) was STc73. STc127 and STc141 were overrepresented and STc95 underrepresented in pneumonia isolates compared with bacteremia isolates. Pneumonia isolates carried higher proportions of virulence genes *sfa/foc*, *papGIII*, *hlyC*, *cnf1*, and *iroN* compared with bacteremia isolates. Virulence factor gene content and antimicrobial drug resistance were higher in pneumonia than in commensal isolates. Genomic and phylogenetic characteristics of *E. coli* pneumonia isolates from critically ill patients indicate that they belong to the extraintestinal pathogenic *E. coli* pathovar but have distinguishable lung-specific traits.

Nosocomial infections remain a major threat for patients and a burden on healthcare institutions, hampering the public health economy. In intensive care units (ICUs), the most common life-threatening nosocomial infection is ventilator-associated pneumonia; the attributable mortality rate is ≈13%, partly because of increased durations of mechanical ventilation and ICU stays (*1*,*2*), all of which generate considerable additional costs (*3*).

Until the early 2000s, *Enterobacteriaceae* were not considered as major pathogens responsible for ventilator-assisted pneumonia (*2*); as such, pathophysiological studies focused mainly on *Pseudomonas aeruginosa*, *Staphylococcus aureus*, and *Acinetobacter baumannii*. However, recent data have consistently shown that *Enterobacteriaceae* are now frequent etiologic agents of ventilator-assisted pneumonia, more frequent than *P. aeruginosa* and *S. aureus* (*4**–**6*). According to the World Health Organization, *Enterobacteriaceae*, including *Escherichia coli*, are among the critical priority antibiotic-resistant bacteria (*7*). Therefore, to optimize patient management, in-depth epidemiologic knowledge of the phenotypic and genotypic characteristics of these bacteria is warranted. Although most *E. coli* responsible for symptomatic extraintestinal infections (*8*), including the urinary tract (*9*), bloodstream (*10*,*11*), cerebral spinal fluid (*12*,*13*), and peritoneum (*14*), have been extensively studied, less is known about *E. coli* strains responsible for pneumonia (*15*), especially in the context of highly virulent and resistant clones such as sequence type (ST) 69 and ST131 (*16*).

Our previous monocentric prospective study in France found a predominance of B2 phylogenetic group (66%) among *E. coli* pneumonia isolates, one third of which belonging to ST complex (STc) 127 (*15*). To obtain further insights in the physiopathology of ventilator-assisted pneumonia, we conducted a multicentric prospective epidemiologic study of genomic and phylogenetic characteristics of *E. coli* strains responsible for pneumonia across France.

## Materials and Methods

### Patients and *E. coli* Isolates

We conducted this prospective study in 14 ICUs throughout France, in collaboration with their hospital laboratories. We selected these ICUs to guarantee appropriate geographic coverage of metropolitan France. The same geographic selection was also applied to the ICUs in the Paris area. The ethics committee of the French Intensive Care Society approved the study (SRLF-CE 12-388), which was registered at ClinicalTrials.gov (NCT03303937). Formal consent was not required because of the observational, noninterventional study design (no change in practices, and all procedures already routinely performed). Patients or family/relatives were informed of the nature of the study and its purpose and objectives and had the option of declining participation.

During a 38-month period (2012–2014), any *E. coli* isolate responsible for pneumonia in a mechanically ventilated patient was collected, regardless of the method of sampling (quantitative cultures of tracheal suctioning, bronchoalveolar lavage, or protected telescoping catheter). Demographic and clinical data for the patient were recorded. We defined ventilator-assisted pneumonia as pneumonia occurring >48 hours after initiation of invasive ventilation, hospital-acquired pneumonia as pneumonia occurring >48 hours after hospital admission but within the first 48 hours of invasive ventilation, and community-acquired pneumonia as pneumonia that occurred either before or within the first 48 hours of hospitalization (*17*). Each *E. coli* isolate was stored at –80°C in brain–heart infusion broth containing glycerol 20% (the COLOCOLI collection).

### *E. coli* Phylotyping, O-Typing, and Virulence Factor Gene Content Determination

We used quadruplex PCR to determine the *E. coli* phylogenetic group (A, B1, B2, C, D, E, F) or belonging to clade I (*18*). Among the strains, we determined the B1 clonal complex 87 (CC87) (Institut Pasteur MLST schema nomenclature corresponding to the ST58 and 155 in the Achtman schema [*19*]), the 10 main B2 subgroups (*20*), and the clonal group A (clonal group A) from the D phylogroup (*21*). The exhaustive correspondence between this typing approach and STc membership according to the currently used Achtman MLST schema is available in (*22*).

We used PCR to determine the most anticipated serotypes in isolates from patients with extraintestinal infections (Table 1) (*23*). Multiplex PCR was used to detect genes encoding for 11 frequently encountered extraintestinal virulence factors (*sfa/foc*, *papC*, *papGII*, *papGIII*, *fyuA*, *iroN*, *aer*, *traT*, *neuC*, *hlyC*, and *cnf1*), which belong to the main classes of virulence factors (adhesins, toxins, iron acquisition systems, and protectins) (*11*). For each isolate, we calculated the virulence score, which was defined by the number of virulence factors present among the 11 tested.

**Table 1:** Characterization of the main *Escherichia coli* B2 phylogroup clones, by combination of subgroup and O-type, among patients with extraintestinal infections, France, 2012–2014*

Subgroup and O-type	B2 clones, no. (%), n = 155
I-O25b	15 (62.5)
I-O6a	2 (8.3)
I-O16	3 (12.5)
II-O22	2 (5)
II-O2b	4 (10)
II-O6a	26 (65)
III-O6a	16 (100)
IV-O2b	19 (95)
VI-O4	13 (92.9)
VII-O18	2 (66.6)
VII-O75	1 (33.3)
IX-O1	7 (50)
IX-O18	3 (21.4)
IX-O2a	3 (21.4)

*Data are presented as no. (%) of each clone within the corresponding subgroup. Roman numerals correspond to the B2 subgroup. The correspondence with the Achtman multilocus sequence type schema is as follows: subgroup I, sequence type complex (STc) 131; subgroup II, STc73; subgroup III, STc127; subgroup IV, STc141; subgroup VI, STc12; subgroup VII, STc14; subgroup IX, STc95 (*22*). The most anticipated serotypes in extraintestinal infections were searched by PCR: O1, O2a, O2b, O4, O6, O7, O12, O15, O16, O17, O18, O22, O25a, O25b, O45a, O75, O78.

### Antimicrobial Resistance Phenotypes

We determined the antimicrobial susceptibility of each isolate by using the disk-diffusion method according to the French Society of Microbiology (https://www.sfm-microbiologie.org) (Table 2). Resistance score was defined as the sum of inactive in vitro antimicrobial agents for each isolate. A score of 1 indicates resistant; 0.5, intermediary; and 0, sensitive. A higher score indicates a more resistant isolate. Detection of gene sequences coding for the TEM, SHV, and CTX-M enzymes was performed by PCR with genomic DNA. The oligonucleotide primer sets specific for the β-lactamase genes used in the PCR assays have been published (*24*,*25*).

**Table 2:** Resistance and virulence traits of the 260 *Escherichia coli* isolates responsible for pneumonia in patients receiving mechanical ventilation, according to phylogenetic group, France, 2012–2014*

Trait	Phylogenetic group		p value	Phylogenetic group				
	B2, n = 155	Non-B2, n = 105		A, n = 22	B1, n = 26	C, n = 20	D, n = 25	F, n = 11
*iroN*	130 (83.9)	52 (49.5)	<0.0001	10 (45.4)	13 (50)	16 (80)	6 (24)	6 (54.5)
*sfa/foc*	109 (70.3)	0	<0.0001	0	0	0	0	0
*neuC*	41 (26.4)	0	<0.0001	0	0	0	0	0
*fyuA*	152 (98.1)	51 (48.6)	<0.0001	7 (31.8)	9 (34.6)	16 (80)	10 (40)	8 (72.7)
*hlyC*	98 (63.2)	2 (1.9)	<0.0001	0	2 (7.7)	0	0	0
*cnf1*	91 (58.7)	1 (1)	<0.0001	0	1 (3.8)	0	0	0
*aer*	65 (41.9)	67 (63.8)	0.0006	14 (63.6)	15 (57.7)	14 (70)	15 (60)	8 (72.7)
*papC*	100 (64.5)	27 (25.7)	<0.0001	7 (31.8)	3 (11.5)	8 (40)	8 (32)	1 (9.1)
*papGII*	27 (17.4)	4 (3.8)	0.0007	0	0	0	4 (16)	0
*papGIII*	64 (41.3)	0	<0.0001	0	0	0	0	0
*traT*	70 (45.2)	82 (78.1)	<0.0001	15 (68.2)	20 (76.9)	17 (85)	20 (80)	9 (81.8)
Virulence score, median (IQR)†	7 (5–7)	3 (2–4)	<0.0001	2.5 (2–4)	3 (1–4)	4 (3–5)	3 (2–3)	3 (2.5–3)
Antimicrobial resistance								
Amoxicillin	75 (48.4)	83 (79)	<0.0001	19 (86.4)	19 (73)	16 (80)	19 (76)	10 (90.9)
Amoxicillin/clavulanic acid	66 (42.6)	68 (64.8)	0.0006	18 (81.8)	13 (50)	15 (75)	14 (56)	8 (72.7)
Piperacillin/tazobactam	21 (13.5)	27 (25.7)	0.02	6 (27.3)	6 (23.1)	7 (35)	3 (12)	5 (45.4)
Cefotaxime	11 (7.1)	17 (16.2)	0.02	4 (18.2)	4 (15.4)	2 (10)	3 (12)	4 (36.4)
Ceftazidime	12 (7.7)	17 (16.2)	0.04	4 (18.2)	4 (15.4)	2 (10)	3 (12)	4 (36.4)
Imipenem	0	1 (1)	0.4	0	1 (3.8)	0	0	0
Gentamicin	4 (2.6)	10 (9.5)	0.02	3 (13.6)	1 (3.8)	2 (10)	1 (4)	3 (27.3)
Amikacin	3 (1.9)	1 (1)	0.6	0	0	0	0	1 (9.1)
Ofloxacin	15 (9.7)	28 (26.7)	0.0005	7 (31.8)	6 (23.1)	6 (30)	2 (8)	6 (54.5)
Ciprofloxacin	13 (8.4)	24 (22.9)	0.002	7 (31.8)	5 (19.2)	6 (30)	2 (8)	4 (36.4)
Resistance score, median (IQR)‡	1.5 (0–4)	4.5 (2.5–7)	<0.0001	5 (3.5–8)	4 (1–6)	4.5 (3.5–7.5)	4 (1.5–5.5)	7.5 (5–9)
ESBL phenotype	10 (6.4)	12 (11.4)	0.2	4 (18.2)	1 (3.8)	1 (4)	2 (8)	3 (27.3)
WT phenotype	84 (54.2)	24 (22.9)	<0.0001	4 (18.2)	7 (26.9)	6 (24)	7 (28)	1 (9.1)

*Values are no. (%) except as indicated. ESBL, extended-spectrum beta-lactamase; IQR, interquartile range; WT, wild type (susceptible to all tested antimicrobials).  †Virulence score was calculated, defined by the number of present virulence factors among the 11 tested. ‡Resistance score was defined as the sum of inactive in vitro antimicrobial agents for each isolate. Tested antimicrobials were gentamicin, amikacin, minocycline, nalidixic acid, ofloxacin, ciprofloxacin, fosfomycin, furans, trimethoprim, amoxicillin, amoxicillin-clavulanic acid, ticarcillin, piperacillin, piperacillin-tazobactam, imipenem, cefotaxime, and ceftazidime. A score of 1 was attributed for a resistant, 0.5 for an intermediary, and 0 for a sensitive isolate; a higher score thus indicated a more resistant isolate. For each antimicrobial, resistance is defined by the sum of resistant or intermediary isolates.

### Characteristics of Other *E. coli* Strain Collections

To learn more about *E. coli* pneumonia strains, we compared the isolates from the COLOCOLI collection with those of 2 published collections, originating from the Paris area in France. In 2010, a total of 280 *E. coli* strains were isolated from fecal samples from community adults and can be considered as commensal strains (COLIVILLE collection) (*26*). In 2005, a total of 373 *E. coli* strains were isolated from the blood of 373 in-patients in 14 hospitals during the course of bacteremia (COLIBAFI collection) (*27*). Of note, 20.6% of isolates from patients with bacteremia were nosocomial and 57% were of urinary origin. Among patients with bacteremia, the portal of entry was pulmonary for <2% (most patients were not in an ICU).

For these strains, we determined the phylogroup/subgroup membership, the presence of the 11 virulence factors cited above, and the susceptibility to 6 antimicrobial drugs (amoxicillin, amoxicillin/clavulanic acid, cefotaxime, amikacin, ofloxacin, and cefoxitin). For all strains, we calculated a virulence score.

### Statistical Analyses

For our analyses we used GraphPad Prism7 software (https://www.graphpad.com) For quantitative variables, results are presented as the median and range, and for categorical variables, as frequency and proportion. Variables were compared according to whether they were nosocomial or community isolates and whether they were of phylogenetic group B2 or not B2. As we compared the virulence factor gene content of the 3 collections, we also compared the proportion of phylogenetic groups and subgroups and of resistant strains. We used the Student *t* test to compare continuous variables and the Fisher exact test to compare categorical variables. Because of the observational design of the study and its exploratory aim, we did not adjust for multiple testing (*28*). We considered p<0.05 to be significant.

## Results

### Host Characteristics

During the study period, we collected 260 *E. coli* isolates from 243 patients with a median age of 64 years (interquartile range 52–73 years) (Table 3). Of these isolates, 117 were responsible for ventilator-assisted pneumonia, 61 for hospital-acquired pneumonia, and 82 for community-acquired pneumonia. The main reasons for ICU admission were acute respiratory failure (n = 61, 25.1%), coma (n = 48, 19.8%), and septic shock (n = 44, 18.1%). A total of 98 (40.3%) patients had received antimicrobial drugs in the previous 3 months.

**Table 3:** Demographics and clinical characteristics of 243 pneumonia patients requiring mechanical ventilation, from whom *Escherichia coli* was isolated, France, 2012–2014*

Characteristic	Value
Age, y, median (IQR)	64 (52–73)
Sex	
M	183 (75.3)
F	60 (24.7)
SAPS II at admission, median (IQR)	57 (42–69)
Comorbid conditions	
Chronic alcohol consumption	56 (23)
Diabetes mellitus	45 (18.5)
Neoplastic disease	43 (17.7)
Immunosuppression†	77 (31.7)
Cirrhosis	12 (4.9)
Chronic kidney disease	18 (7.4)
Dialysis	5 (2.1)
Chronic respiratory disease	33 (13.6)
Chronic heart failure	43 (17.7)
Reason for ICU admission	
Acute respiratory failure	61 (25.1)
Coma	48 (19.8)
Septic shock	44 (18.1)
Cardiac arrest	28 (11.5)
Cardiogenic shock	14 (5.8)
Polytrauma	22 (9.1)
Postoperative care	8 (3.3)
Hemorrhagic shock	5 (2.1)
Exposure to antimicrobial drug therapy in previous 3 mo	98 (40.3)
Amoxicillin	6 (2.5)
Amoxicillin/clavulanic acid	38 (15.6)
Third-generation cephalosporin	19 (7.8)
Aminoglycosides	29 (11.9)
Piperacillin/tazobactam	24 (9.9)
Quinolone	10 (4.1)
Carbapenem	11 (4.5)
Polymicrobial sampling	57 (23.5)
ICU length of stay, d (IQR)	17 (7–33)
Hospital length of stay, d (IQR)	24 (10–45)
Death	
While in ICU	90 (37)
While in hospital	99 (40.7)
Associated with *E. coli*	27 (11.1)

*Values are no. (%) except as indicated. ICU, intensive care unit; IQR, interquartile range; SAPS II, Simplified Acute Physiology Score II.  †Defined by >1 immunosuppression factor among neoplastic disease, hematologic malignancy, HIV infection, immunosuppressive therapy, corticosteroids therapy.

### *E. coli* Characteristics

*E. coli* alone was isolated from 76.5% of the respiratory samples, whereas 23.5% of the samples were polymicrobial. The monomicrobial and polymicrobial samples did not differ in terms of phylogroup, virulence factor content, or antimicrobial resistance. We compiled classifications of the different phylogenetic groups/subgroups (Table 4) and details about their community or nosocomial status (Appendix Table 1). The main phylogenetic groups were B2 (n = 155, 59.6%), B1 (n = 26, 10%), and D (n = 25, 9.6%). The most commonly identified lineages were STc73 (subgroup II, n = 40, 25.8% of B2 isolates), STc131 (subgroup I, n = 24, including 18 ST131), STc69 (clonal group A [*29*], n = 20), STc141 (subgroup IV, n = 20), and STc127 (subgroup III, n = 16). STc95 (subgroup IX) encompassed 14 strains (9% of B2 isolates). Community and nosocomial isolates did not differ in terms of phylogenetic group, except for C phylogroup isolates, which had a community predisposition (14.6% community vs. 4.5% nosocomial; p = 0.01).

**Table 4:** Phylogenetic groups/subgroups of *Escherichia coli* isolated from 260 pneumonia patients requiring mechanical ventilation, France, 2012–2014*

Phylogroup	No. (%)
A	22 (8.5)
B1	26 (10)
CC87†	11 (42.3)
Non-CC87	15 (57.7)
B2	155 (59.6)
I ST131†	18 (11.6)
I non-ST131	6 (3.9)
II	40 (25.8)
III	16 (10.3)
IV	20 (12.9)
V	1 (0.6)
VI	14 (9)
VII	3 (1.9)
IX	14 (9)
Unassigned	23 (14.8)
C	20 (7.7)
D	25 (9.6)
CGA†	20 (80)
Non-CGA	5 (20)
F	11 (4.2)
Clade I	1 (0.4)

*Nosocomial isolates encompass ventilator-associated pneumonia and hospital-acquired pneumonia isolates. CC87, clonal complex 87 (sequence type complex [STc] 155) (*19*); CGA, clonal group A (STc69) (*21*); ST131, sequence type 131.  The correspondence with the Achtman MLST schema is as follows: subgroup I, STc131; subgroup II, STc73; subgroup III, STc127; subgroup IV, STc141; subgroup V, STc144; subgroup VI ,STc12; subgroup VII, STc14; subgroup IX, STc95. No isolate belonged to subgroup VIII (STc452), X (STc372), or E phylogroup (*22*). †Proportions of subgroups are reported as fractions of the respective phylogroups.

We identified the O-type of 163 strains. We identified B2 phylogroup strains at the clonal level as having a combination of subgroup and O-type, as previously described (*27*) (Table 1). Among B2 strains, clones II-O6a were predominant, followed by IV-O2b, I-O25b (which belongs to ST131), III-O6a (which belongs to the highly virulent archetypal strain 536 [*30*]), and VI-O4.

Resistance and virulence traits of the 260 strains are detailed in Table 2 and Appendix Table 2. B2 phylogroup strains carried more virulence factor genes (virulence score 7 [5–7]) than non–B2 phylogroup strains (3 [2–4]; p<0.0001). However, *traT* genes were significantly more present in non-B2 (78.1%) than B2 phylogroup (45.2%) isolates (p<0.0001), as were *aer* genes (non-B2 63.8% and B2 41.9%; p = 0.0006). In nearly three quarters of strains, mainly B2 strains, we found *iroN* (70%) and *fyuA* (78.1%). Community and nosocomial isolates did not differ in terms of virulence and resistance scores. A total of 22 (8.5%) isolates were producers of extended-spectrum β-lactamases, including 13 CTX-M-1 group, 6 CTX-M-9 group, and 3 TEM. One isolate produced an OXA-48 carbapenemase, 28 (10.8%) isolates were resistant to cefotaxime, and 48 (18.5%) strains were resistant to piperacillin-tazobactam. B2 phylogroup strains were more sensitive to antimicrobials (resistance score 1.5 [0–4]) than were non-B2 phylogroup strains (resistance score 4.5 [2.5–7]; p<0.0001).

### General Comparisons of the COLOCOLI, COLIBAFI, and COLIVILLE Collections

When comparing the pneumonia *E. coli* isolates with those from the 2 other collections, we found strong differences (Table 5; Figure). The B2 phylogroup was overrepresented in pneumonia strains (59.6 %) compared with commensal strains (32.1%; p<0.0001) but not with bacteremia strains (52%; p = 0.06). Among B2 phylogroup pneumonia strains, subgroup III (STc127) was significantly overrepresented (10.3%) compared with the 2 other collections (bacteremia 4.1%, p = 0.03; commensal 2.2%, p = 0.02). The proportion of subgroup IV (STc141) isolates was significantly higher among pneumonia strains (12.9%) than bacteremia strains (2.6%; p = 0.0002), whereas subgroup IX (STc95) isolates (9%) were underrepresented compared with the bacteremia B2 phylogroup strains (29.4%; p<0.0001). Within the D phylogenetic group, the proportions of clonal group A pneumonia isolates (80%) were greater than those of commensal isolates (40%; p = 0.001).

**Table 5:** Proportion of phylogenetic groups, B2 subgroups, D CGA, and B1 CC87 among pneumonia, bacteremia, or commensal isolates

Phylogenetic characteristics	Pneumonia isolates	Bacteremia isolates	Commensal isolates
A	–	+	++
B1	+	–	+
CC87†	+++	+++	+
B2	++++	++++	+++
I	+	+	+
II	++	++	++
III	+	–	–
IV	+	–	+
IX	–	++	+
C	–	–	–
D	–	+	+
CGA*	++++	++++	+++
E	–	–	–
F	–	–	–

*CGA, clonal group A (sequence type complex [STc] 69) (*21*); CC87, clonal complex 87 (STc155) (*19*); –, <10%; +, 10%–20%; ++, 20%–30%; +++ 30–50%; ++++, >50% of isolates. Correspondence with the Achtman multilocus sequence typing schema is subgroup I = STc131; subgroup II = STc73; subgroup III = STc127; subgroup IV = STc141; and subgroup IX = STc95 (*22*). †Proportions of subgroups are calculated within each respective phylogroup.

**Figure:**
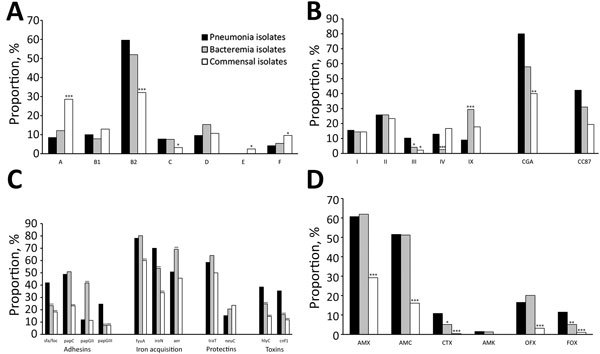
Comparison of *Escherichia coli* isolates among pneumonia patients with extraintestinal infections, France, 2012–2014, with commensal *E. coli* (COLIVILLE collection) and *E. coli* bacteremia isolates (COLIBAFI collection). A) Proportions of *E. coli* phylogenetic groups and subgroups; B) B2 subgroups, D CGA, and B1 CC87; C) virulence factors; and D) antimicrobial drug resistance. Roman numerals correspond to the B2 subgroup. Correspondence with the Achtman multilocus sequence typing schema is as follows: subgroup I, STc131; subgroup II, STc73; subgroup III, STc127; subgroup IV, STc141; subgroup VI, STc12; subgroup VII, STc14; subgroup IX, STc95 (*22*). Proportions of subgroups are reported as fractions of the respective phylogroups. Asterisks indicate a significant difference between respiratory isolates and strains responsible for bacteremia (COLIBAFI collection) or commensal strains (COLIVILLE collection): *p<0.05; **p<0.005; ***p<0.0005. AMC, amoxicillin/clavulanic acid; AMK, amikacin; AMX, amoxicillin; CTX, cefotaxime; FOX, cefoxitin; OFX, ofloxacin; STc, sequence type complex.

Virulence scores of pneumonia isolates (5 [3–7]) were significantly higher than those of commensal isolates (3 [1–5]; p<0.0001) but not different from those of the bacteremia isolates (4 [2–7]; p = 0.3). However, some adhesins (*sfa/foc*, *papGIII*), some toxins (*hlyC*, *cnf1*), and *iroN* were significantly overrepresented in pneumonia *E. coli* strains compared with strains in the 2 other collections. 

Pneumonia *E. coli* isolates were more resistant than commensal isolates to all tested antimicrobial drugs except amikacin. Pneumonia isolates and bacteremia isolates did not differ in terms of antimicrobial drug susceptibility, except for resistance to cefotaxime (pneumonia isolate resistance 10.8%) and cefoxitin (pneumonia isolate resistance 11.5%) compared with bacteremia isolates (5.1%; p = 0.009 for cefotaxime, p = 0.004 for cefoxitin). Of note, when the 260 pneumonia isolates were compared with the 220 bacteremia strains of urinary tract origin, they were still distinguishable in terms of phylogroups/subgroups and virulence factor content (Appendix).

## Discussion

This prospective nationwide study provides data on *E. coli* pneumonia isolates in critically ill patients. With regard to the characteristics of *E. coli* pneumonia isolates, we found the following: 1) a preponderance of phylogenetic group B2 (59.6%); 2) a predominant STc73 (subgroup II) lineage and threatening emergence of ST131 (within subgroup I), STc69 (clonal group A ), and STc127 (subgroup III), along with STc141 (subgroup IV); 3) a much lower proportion of STc95 (subgroup IX) in B2 pneumonia than in bacteremia isolates; 4) a specific virulence factor gene content in pneumonia versus bacteremia strains. Taken together, these epidemiologic, phylogenetic, genotypic, and experimental data argue for inclusion of *E. coli* pneumonia isolates in the extraintestinal pathogenic *E. coli* (ExPEC) pathovar but with distinguishable lung-specific traits.

In 2010, Croxen and Finlay reviewed the molecular mechanisms of *E. coli* pathogenicity (*31*). Among ExPEC, numerous pathovars were listed, including uropathogenic and neonatal meningitis pathogenic *E. coli*, but none concerned the lungs. Pneumonia was not even cited as a possible disease caused by *E. coli*.

The situation is now clearly different. In 2012, Hamet et al. reported that *Enterobacteriaceae* accounted for a quarter of the 323 episodes of ventilator-assisted pneumonia occurring in their ICU (*6*). Our group showed that over a 5-year analysis of ventilator-assisted pneumonia episodes, finding *Enterobacteriaceae* as the responsible pathogen increased significantly (*5*). In an international multicenter study, Kollef et al. also confirmed that *Enterobacteriaceae* were the leading pathogens of ventilator-assisted pneumonia in the ICU (*4*). Among them, *E. coli* is a major threat, recently highlighted by the World Health Organization, because of its ever-increasing resistance to antimicrobial drugs (*7*).

ExPEC are characterized by pathogenic virulence factor genes coding for various combinations of adhesins, toxins, iron-acquisition systems, capsule production, and toxins that enable them to cause disease once outside the host gut reservoir (*32*). ExPEC virulence factors are encoded on the bacterial chromosome, where they are usually located within pathogenicity-associated islands (PAIs) or plasmids. Most ExPEC isolates belong to the B2 phylogroup and, to a lesser extent, the D phylogroup. More in-depth analysis of ExPEC strains has enabled characterization of particular STs of ExPEC isolates including ST131, ST73, and ST127 (*33*). Our most striking finding was the specificity of pneumonia *E. coli* strains, compared with bacteremia ones, within the ExPEC family (Table 5). First, although B2 phylogroup strains are preponderant in ExPEC (*15*,*34*), we found a trend toward an even greater proportion of B2 isolates among pneumonia isolates (59.6%) than among bacteremia isolates, whatever the origin (52%; p = 0.06) (Table 3; Figure). This finding is in the range of what is observed in urosepsis isolates (62%) (*11*) but a little less than in neonatal meningitis strains (68%) (*13*). Then, among B2 phylogroup isolates (Table 4), if the predominance of subgroup II (STc73) was expected, we found high proportions of specific phylogenetic group B2 clones among other lineages, which could be worrisome (Table 1). The ST131 O25b:H4 clone represented 9.7% of the B2 phylogroup isolates, 53.3% of them producing an extended-spectrum β-lactamase. Subgroup IV (STc141) was significantly more present among pneumonia strains than among bacteremia strains. Challenging the hypothesis of a commensal character, with a low level of human invasiveness (*27*), this finding indicates that subgroup IV isolates may have a high affinity for the respiratory tract. Last, we must highlight the greater proportion of subgroup III among pneumonia isolates (namely STc127) compared with bacteremia and commensal isolates, in agreement with previous findings from our group (*15*). Contrary to isolates from the other 2 collections, pneumonia isolates were composed of fewer IX subgroup strains (STc95). Whereas subgroup IX is usually well represented among commensal and other pathogenic *E. coli* strains (*26*,*27*,*35*), these data suggest that the respiratory tract is less suitable than other tissues for subgroup IX implantation, a finding in agreement with our previous report in which subgroup IX was not represented (*15*).

Among D phylogroup strains, the higher prevalence of clonal group A (STc69) in pneumonia isolates than in bacteremia and commensal isolates was unexpected. Among 571 D phylogroup *E. coli* responsible for extraintestinal infection, Johnson et al. reported only 144 clonal group A *E. coli* (25.2%) (*29*). The multidrug-resistant nature of these pathogens is of increasing concern (*29*,*36*).

Regarding virulence factor gene content, the literature suggests that PAIs involved in ExPEC causing pneumonia differ from those involved in urinary tract and bloodstream infections (*37*,*38*). Using PAI deletion mutants in a rat model of pneumonia, Phillips-Houlbracq et al. related pneumonia pathogenicity to the presence of PAIs I and III (*37*). In our study (Figure), pneumonia isolates differed from bacteremia isolates because they significantly more often carried *sfa/foc*, *iroN* (both belonging to PAI III), *papGIII* and *cnf1* (belonging to PAI II), and *hlyC* (belonging to PAI I and II). The role of α-hemolysin in experimentally induced pneumonia in rats has been reported (*15*,*39*). Of note, the virulence factor content of the pneumonia strains still differs when we consider only the urosepsis isolates (Appendix). Although additional studies are required to confirm, these findings do suggest a coherent molecular trait for the isolates’ lung specificity.

Consistent with data in the literature (*15*), we found that pneumonia B2 isolates were less resistant to antimicrobial drugs (B2 resistance score 1.5 [0–4] vs. non-B2 resistance score 4.5 [2.5–7]; p<0.0001) but carried more virulence factor genes (B2 virulence score 7 [5–7] vs. non-B2 virulence score 3 [2–4]; p<0.0001) (Table 2). However, contrary to an old belief, this trade-off does not mean that antimicrobial drug resistance decreases with increasing virulence (*40*). Indeed, virulence and antimicrobial-drug resistance were both higher in pneumonia isolates than in commensal isolates and the following were more highly represented: *sfa/foc*, *papC*, *papGIII*, *fyuA*, *iroN*, *hlyC*, and *cnf1*.

Our study should be interpreted within the context of its limitations. First, although our collection of *E. coli* pneumonia isolates is large, results regarding some subgroup analyses will require confirmation because of their sample size. Our choice of PCR rather than whole-genome characterization was governed by our wish to compare the 2 other collections with the same type of data. Despite these limitations, our analysis based on 14 centers representing France on a population and geographic level, its prospective design, and the comparison of our large number of isolates with those from recently published collections enable us to draw valid conclusions. We did not assess the functionality and expression of the encoded virulence factors in these isolates. Our team has consistently demonstrated these features in several murine models of infection (including pneumonia) and observed a strong correlation between the presence of these genes and death (*15*,*34*,*37*).

Our data raise the question of why certain clonal lineages were overrepresented in patients with respiratory tract infection. Patients acquire *E. coli* infection from their own digestive tract (*41*). This event implies an upward retrograde motion of the bacterial cells to reach the oropharynx and the lung parenchyma and suggests particular metabolic-adaptation and response-to-stress characteristics (e.g., to overcome the acidity of the stomach). We have previously shown that some *E. coli* strains are capable of high growth capacities in relation to metabolic pathways (*42*) while others are highly resistant to stress (*43*). For both studies, however, no link to specific clones could be established. The specific organ tropism therefore more probably results from a combination of genetic background and virulence factors.

In summary, we identified emerging pneumonia-causing pathogenic *E. coli* whose main characteristics define them as ExPEC. Their specificities include a very strong proportion of B2 phylogroup isolates; a high proportion of subgroups II (STc73), I (STc131), IV (STc141), and III (STc127); and consequent proportions of clonal group A (STc69) isolates within the D phylogroup. Virulence factor gene content of pneumonia isolates also appeared to be singular compared with that of bacteremia isolates, among them urosepsis isolates. These epidemiologic data underline the specificity of pneumonia *E. coli* populations and may help with the design of more targeted therapies.

Appendix. Supplemental results for study of Pneumonia-Specific Escherichia coli isolates with distinct phylogenetic and virulence profiles, France, 2012–2014
