# Supplementary material for: Pneumonia-Specific Escherichia coli with Distinct Phylogenetic and Virulence Profiles, France, 2012–2014
Source: Emerg Infect Dis. 2019 Apr;25(4):710–8. doi: 10.3201/eid2504.180944 (PMC6433033; doi:10.3201/eid2504.180944)
Supplement: Supplementary file 1 — Appendix. Supplemental results for study of Pneumonia-Specific Escherichia coli isolates with distinct phylogenetic and virulence profiles, France, 2012–2014 [file 18-0944-Techapp-s1.pdf]

Article DOI: https://doi.org/10.3201/eid2504.180944

Pneumonia-Specific *Escherichia coli* Isolates with Distinct Phylogenetic and Virulence Profiles, France, 2012–2014

Appendix

Results

Appendix Table 1. *E. coli* phylogenetic groups belonging according to the community or nosocomial status*

Phylogroups	All (n = 260)	Community isolates (n = 82)	Nosocomial isolates (n = 178)	p value
A	22 (8.5)	5 (6.1)	17 (9.6)	0.5
B1	26 (10)	4 (4.8)	22 (12.4)	0.08
CC87†	11 (42.3)	2 (50)	9 (40.9)	1
Non-CC87	15 (57.7)	2 (50)	13 (59.1)	
B2	155 (59.6)	54 (65.9)	101 (56.7)	0.2
I ST131†	18 (11.6)	7 (13)	11 (10.9)	0.8
I non-ST131	6 (3.9)	3 (5.6)	3 (3)	0.4
II	40 (25.8)	14 (26)	26 (25.7)	1
III	16 (10.3)	5 (9.3)	11 (10.9)	1
IV	20 (12.9)	4 (7.4)	16 (15.8)	0.2
V	1 (0.6)	1 (1.9)	0	0.3
VI	14 (9)	6 (11.1)	8 (7.9)	0.6
VII	3 (1.9)	1 (1.9)	2 (2)	1
IX	14 (9)	4 (7.4)	10 (9.9)	0.8
Unassigned	23 (14.8)	9 (16.7)	14 (13.9)	0.6
C	20 (7.7)	12 (14.6)	8 (4.5)	0.01
D	25 (9.6)	4 (4.9)	21 (11.8)	0.1
CGA†	20 (80)	3 (75)	17 (81)	1
Non-CGA	5 (20)	1 (25)	4 (19)	
F	11 (4.2)	3 (3.7)	8 (4.5)	1
Clade I	1 (0.4)	0	1 (0.6)	1

*Abbreviations: ST131, sequence type 131; CGA, clonal group A (STc69) [*1*] CC87, Clonal complex 87 (STc155) [*18*]. Nosocomial isolates encompass VAP and HAP isolates. Data are presented as n (%).The correspondence with the Achtman MLST schema is as follow: subgroup I = STc131; subgroup II = STc73; subgroup III = STc127; subgroup IV = STc141; subgroup V = STc144; subgroup VI = STc12; subgroup VII = STc14; subgroup IX = STc95. No isolate belonged to subgroup VIII (STc452), X (STc372) or to E phylogroup [*2*].
†Proportions of subgroups are reported as fractions of the respective phylogroups.

Appendix Table 2. Resistance and virulence traits of the 260 *E. coli* isolates, according to the community or nosocomial status*

Trait	All (n = 260)	Community isolates (n = 82)	Nosocomial isolates (n = 178)	p value
iroN	182 (70)	60 (73.2)	122 (68.5)	0.5
sfa/foc	109 (41.9)	38 (46.3)	71 (39.9)	0.3
neuC	41 (15.2)	12 (14.6)	29 (16.3)	0.9
fyuA	203 (78.1)	70 (85.4)	133 (74.7)	0.06
hlyC	100 (38.5)	35 (42.7)	65 (36.5)	0.4
cnf1	92 (35.4)	32 (39)	60 (33.7)	0.4
aer	132 (50.8)	38 (46.3)	94 (52.8)	0.3
papC	127 (48.8)	35 (42.7)	92 (51.7)	0.2
papGII	31 (11.9)	8 (9.8)	23 (12.9)	0.5
papGIII	64 (24.6)	19 (23.2)	45 (25.3)	0.8
traT	152 (58.5)	47 (57.3)	105 (59)	0.9
Virulence score	5 [3-7]	5 [3-7]	5 [3-7]	0.8
Resistance score	3.5 [0-5.5]	3 [0-5]	3.5 [0-5.5]	0.3
ESBL phenotype	22 (8.5)	4 (4.9)	18 (10.1)	0.2
WT phenotype	108 (41.5)	38 (46.3)	70 (39.3)	0.3

*Abbreviations: ESBL, extended-spectrum beta-lactamase; WT phenotype, Wild type phenotype (susceptibility to all tested antibiotics). Nosocomial isolates encompass VAP and HAP isolates.Data are presented as n (%) or median [interquartile range]. For each antibiotic, antimicrobial resistance is defined by the sum of resistant or intermediary isolates.

Comparison of phylogroups, subgroups, and virulence factor gene content of pneumonia isolates *vs* bacteraemia isolates of urinary origin

A former study from our group showed, among strains responsible for bacteremia, differences in the bacteremia strains of urinary tract origin with the presence of specific clones, whereas the bacteraemia strains of digestive origin resembled more the commensal strains (*3*).

Hence, we sought to compare *E. coli* pneumonia isolates and bacteraemia isolates of urinary origin (Appendix Table 3). Similarities lay in the phylogroup as well as in the subgroup distribution with two noticeable exceptions: subgroup IV was clearly over-represented in pneumonia isolates whereas this was the other way round for subgroup IX.

Differences were found in the distribution of virulence factor genes more than in the overall virulence score (5 [3-7] for the pneumonia isolates *vs* 4 [2-4] for the bacteremia isolates of urinary origin). Noticeable differences were related to a greater number of pneumonia isolates harbouring *sfa/foc, papGIII* and *cnf1*, and a greater number of bacteremia isolates from urinary origin harbouring *papC, papGII*.

Appendix Table 3. Comparison of phylogroups, subgroups, and virulence factor gene content of pneumonia isolates *vs* bacteraemia isolates of urinary origin*

Variable	Pneumonia isolates (n = 260)	Bacteremia isolates of urinary origin (n = 220)	p value
Phylogroups			
A	8.5	5.5	0.2
B1	10	5	0.06
B2	59.6	63.2	0.5
C	7.7	7.3	1
D	9.6	16.4	0.03
E	0	0	1
F	4.2	2.7	0.5
Subgroups†			
I	15.5	7.9	0.3
II	25.8	27.3	0.8
III	10.3	4.5	0.07
IV	12.9	1.4	0.0001
IX	9	34.5	<0.0001
CGA	80	69.4	0.4
CC87	42.3	27.3	0.5
Virulence factor genes			
sfa/foc	41.9	28.6	0.003
papC	48.8	67.7	<0.0001
papGII	11.9	59.1	<0.0001
papGIII	24.6	8.6	<0.0001
fyuA	78.1	88.2	0.004
iroN	70	62.3	0.08
aer	50.8	70	<0.0001
traT	58.5	70	0.01
neuC	15.2	25.5	0.009
hlyC	38.5	31.8	0.2
cnf1	35.4	20	0.0002

*The correspondence with the Achtman MLST schema is as follow: subgroup I = STc131; subgroup II = STc73; subgroup III = STc127; subgroup IV = STc141; subgroup V = STc144; subgroup VI = STc12; subgroup VII = STc14; subgroup IX = STc95. No isolate belonged to subgroup VIII (STc452), X (STc372) or to E phylogroup [*2*].

†Proportions of subgroups are reported as fractions of the respective phylogroups. Data are presented as n (%).Electronic supplemental material to "Emerging pneumonia-specific *Escherichia coli* isolates with distinct phylogenetic and virulence profiles: a nationwide study"

Results

Appendix Table 4. Phylogenetic groups, subgroups, serotypes, ESBL phenotype and virulence factor gene content of each isolate of the COLOCOLI collection*

Isolate ID	ESBL phenotype	Phylogenetic group	B2 subgroup	D subgroup	B1 subgroup	Serotype	*neuC*	*iroN*	*sfa*	*fyuA*	*hly*	*cnf1*	*aer*	*papC*	*papGII*	*papGIII*	*traT*
AN01	0	B2	III	non-D	non-B1	O6	0	1	1	1	1	1	0	1	0	1	1
AN02	0	B1	non-B2	non-D	CC87	ND	0	1	0	1	0	0	1	1	0	0	1
AN03	1	F	non-B2	non-D	non-B1	ND	0	1	0	1	0	0	1	0	0	0	1
AN04	0	B2	IV	non-D	non-B1	O2b	1	1	1	1	1	1	1	1	0	1	1
AN05	0	B2	IX	non-D	non-B1	O18	1	1	1	1	0	0	1	0	0	0	1
AN06	0	B2	UA	non-D	non-B1	O75	0	1	1	1	1	1	0	1	0	1	0
AN10	0	B2	III	non-D	non-B1	O6	0	0	0	1	0	0	0	1	0	1	0
AN12	0	B2	II	non-D	non-B1	O2b	0	0	0	1	1	1	1	1	1	0	1
AN13	0	D	non-B2	CGA	non-B1	O17	0	1	0	0	0	0	1	1	1	0	1
AN15	0	B1	non-B2	non-D	CC87	ND	0	1	0	0	0	0	1	0	0	0	1
AN16	0	B2	IX	non-D	non-B1	O2a	1	0	0	1	0	0	0	1	1	0	1
AN17	0	B2	II	non-D	non-B1	O6	0	1	1	1	1	1	1	0	0	0	0
AN18	0	C	non-B2	non-D	non-B1	O78	0	1	0	1	0	0	1	0	0	0	1
AN19	0	C	non-B2	non-D	non-B1	ND	0	1	0	1	0	0	1	1	0	0	1
AN20	0	A	non-B2	non-D	non-B1	ND	0	0	0	1	0	0	0	0	0	0	1
AN21	0	B2	UA	non-D	non-B1	ND	0	0	0	0	0	0	0	1	0	0	0
AN22	0	B1	non-B2	non-D	no CC87	ND	0	0	0	0	0	0	0	0	0	0	1
AN24	0	A1	non-B2	non-D	non-B1	ND	0	1	0	1	0	0	1	0	0	0	1
AN25	0	C	non-B2	non-D	non-B1	ND	0	1	0	1	0	0	1	0	0	0	1
AN28	0	B2	IX	non-D	non-B1	O1	1	1	0	1	0	0	1	1	1	0	1
AN30	0	D	non-B2	CGA	non-B1	O25b	0	0	0	1	0	0	0	0	0	0	1
AN33	0	B2	II	non-D	non-B1	O6	0	1	1	1	1	0	0	0	0	0	0
AN34	0	B2	UA	non-D	non-B1	O18	0	1	0	1	1	0	1	1	1	0	1
AN36	1	B2	I	non-D	non-B1	O25b	0	0	0	1	0	0	1	1	0	0	1
AN37	0	B2	II	non-D	non-B1	O6	0	1	1	1	1	1	0	1	0	1	0
AN39	0	B2	IX	non-D	non-B1	O2a	1	1	0	1	0	0	1	1	1	0	1
AN41	0	B2	VI	non-D	non-B1	O4	0	1	1	1	1	1	1	1	1	0	0
AN43	0	D	non-B2	no CGA	non-B1	ND	0	1	0	0	0	0	0	0	0	0	1
AN44	0	C	non-B2	non-D	non-B1	ND	0	1	0	1	0	0	0	0	0	0	1
AN45	0	B2	UA	non-D	non-B1	O75	0	1	1	1	1	1	0	1	0	1	0
AN46	0	B2	II	non-D	non-B1	O6	0	1	1	1	1	1	0	0	0	0	0
AN47	0	B2	II	non-D	non-B1	O6	0	1	1	1	1	1	0	1	0	1	0
AN48	0	B2	II	non-D	non-B1	O6	0	1	1	1	1	1	0	1	0	1	0
AN49	0	C	non-B2	non-D	non-B1	ND	0	1	0	0	0	0	0	0	0	0	1
AVC02	1	B2	I	non-D	non-B1	O25b	0	0	0	1	0	0	1	0	0	0	0
AVC03	1	F	non-B2	non-D	non-B1	ND	0	1	0	1	0	0	1	0	0	0	1
BCH811	0	B2	IV	non-D	non-B1	O2b	0	1	1	1	1	1	0	1	0	1	0
BCH818	0	B2	VII	non-D	non-B1	O18	0	1	1	1	0	0	0	1	1	0	0
BCH826	0	F	non-B2	non-D	non-B1	ND	0	0	0	1	0	0	0	0	0	0	1
BCH830	0	B2	VII	non-D	non-B1	O18	0	1	1	1	1	0	0	1	1	0	0
BCH838	0	F	non-B2	non-D	non-B1	ND	0	1	0	1	0	0	0	0	0	0	1
BCH841	0	F	non-B2	non-D	non-B1	ND	0	0	0	1	0	0	0	0	0	0	1

Isolate ID	ESBL phenotype	Phylogenetic group	B2 subgroup	D subgroup	B1 subgroup	Serotype	*neuC*	*iroN*	*sfa*	*fyuA*	*hly*	*cnf1*	*aer*	*papC*	*papGII*	*papGIII*	*traT*
BCH852	0	B2	IX	non-D	non-B1	O1	1	1	0	1	0	0	1	1	1	0	1
BCH853	0	C	non-B2	non-D	non-B1	ND	0	1	0	1	0	0	0	0	0	0	1
BCH855	0	D	non-B2	no CGA	non-B1	ND	0	0	0	0	0	0	0	0	0	0	0
BCH857-15	0	B2	IX	non-D	non-B1	O1	1	1	0	1	0	0	1	1	1	0	1
BCH857-72	0	B2	UA	non-D	non-B1	O75	0	1	1	1	1	1	0	1	0	1	0
BCH858	0	F	non-B2	non-D	non-B1	ND	0	0	0	1	0	0	1	0	0	0	1
BCH859	0	C	non-B2	non-D	non-B1	ND	0	0	0	1	0	0	1	1	0	0	1
BCH860	0	B2	IX	non-D	non-B1	O1	1	1	0	1	0	0	1	1	1	0	1
BCH861	0	B1	non-B2	non-D	CC87	ND	0	0	0	0	0	0	0	0	0	0	1
BCH862	0	A1	non-B2	non-D	non-B1	ND	0	1	0	0	0	0	0	0	0	0	1
BCH865	0	B2	IX	non-D	non-B1	O1	1	1	0	1	0	0	1	1	1	0	1
BCH869	0	B2	I	non-D	non-B1	O6	0	1	0	1	0	0	0	0	0	0	1
BCH876	0	B1	non-B2	non-D	no CC87	ND	0	0	0	0	0	0	0	0	0	0	0
BCH877	1	B2	I	non-D	non-B1	O16	0	0	0	1	0	0	1	0	0	0	1
BCH885	0	C	non-B2	non-D	non-B1	ND	0	1	0	1	0	0	1	1	0	0	1
BCH887	0	D	non-B2	CGA	non-B1	ND	0	0	0	1	0	0	1	0	0	0	1
BCH892	0	D	non-B2	CGA	non-B1	ND	0	0	0	1	0	0	1	0	0	0	1
BCH898	0	C	non-B2	non-D	non-B1	ND	0	1	0	1	0	0	1	1	0	0	0
BCH902	1	F	non-B2	non-D	non-B1	O2b	0	0	0	0	0	0	1	1	0	0	0
BCH906	0	B2	VII	non-D	non-B1	O75	0	0	0	1	0	0	1	0	0	0	1
BCH907	0	B2	II	non-D	non-B1	O2b	0	1	1	1	1	1	1	1	0	1	0
BCH914	0	B2	I	non-D	non-B1	O16	0	0	0	1	0	0	1	0	0	0	1
BCH916	0	B2	I	non-D	non-B1	O25b	0	0	0	1	0	0	1	0	0	0	0
BCH921	0	B2	III	non-D	non-B1	O6	0	1	1	1	1	0	0	1	0	0	0
BCH924	0	B2	II	non-D	non-B1	O6	0	1	1	1	1	1	0	1	1	0	1
BCH925	0	B2	II	non-D	non-B1	O6	0	1	1	1	1	1	1	1	1	0	1
BCH926-49	0	C	non-B2	non-D	non-B1	ND	0	1	0	1	0	0	1	0	0	0	1
BCH926-55	0	B2	III	non-D	non-B1	O6	0	1	1	1	1	1	0	1	0	1	1
BCH927	0	B2	III	non-D	non-B1	O6	0	1	1	1	1	1	0	1	0	0	0
BCH933	0	B2	III	non-D	non-B1	O6	0	1	1	1	1	1	0	1	0	0	0
BCH935	0	B2	VI	non-D	non-B1	O4	0	1	0	1	1	1	0	1	0	1	1
BCH943	0	D	non-B2	CGA	non-B1	O17	0	1	0	0	0	0	1	1	1	0	1
BCH957	0	B2	UA	non-D	non-B1	O75	0	1	1	1	1	1	0	1	0	1	0
BCH964	0	B1	non-B2	non-D	no CC87	ND	0	0	0	1	0	0	0	0	0	0	1
BDX01	1	A1	non-B2	non-D	non-B1	ND	0	0	0	0	0	0	1	1	0	0	1
BDX02	0	B2	I	non-D	non-B1	O25b	0	1	0	1	0	0	1	1	0	1	1
BDX03	0	D	non-B2	CGA	non-B1	O17	0	1	0	0	0	0	1	0	0	0	1
BDX04	0	A0	non-B2	non-D	non-B1	ND	0	0	0	0	0	0	0	0	0	0	0
BDX07	1	D	non-B2	CGA	non-B1	O25b	0	0	0	0	0	0	1	0	0	0	1
BDX09	0	F	non-B2	non-D	non-B1	ND	0	0	0	1	0	0	1	0	0	0	1
BDX10	0	A	non-B2	non-D	non-B1	ND	0	0	0	1	0	0	1	0	0	0	0
BDX11	0	B2	VI	non-D	non-B1	O4	0	1	1	1	1	1	0	1	0	1	0
BDX13	0	B2	II	non-D	non-B1	O6	0	1	1	1	1	1	0	1	0	1	0
BDX14	0	B2	II	non-D	non-B1	O4	0	1	1	1	0	0	0	0	0	0	1
BDX15	0	C	non-B2	non-D	non-B1	ND	0	0	0	0	0	0	0	0	0	0	0
BDX18	1	D	non-B2	CGA	non-B1	ND	0	0	0	1	0	0	0	0	0	0	1
BDX19	1	B2	I	non-D	non-B1	O25b	0	0	0	1	1	1	1	1	1	0	1
BDX20	0	C	non-B2	non-D	non-B1	ND	0	1	0	1	0	0	1	1	0	0	1

Isolate ID	ESBL phenotype	Phylogenetic group	B2 subgroup	D subgroup	B1 subgroup	Serotype	*neuC*	*iroN*	*sfa*	*fyuA*	*hly*	*cnf1*	*aer*	*papC*	*papGII*	*papGIII*	*traT*
BDX21	0	B1	non-B2	non-D	no CC87	ND	0	0	0	0	0	0	1	1	0	0	1
BDX22	1	B2	I	non-D	non-B1	O25b	0	0	0	1	0	0	1	0	0	0	0
BDX23	0	B2	II	non-D	non-B1	ND	0	1	1	1	0	0	1	0	0	0	1
BDX25	0	B2	UA	non-D	non-B1	O18	0	0	0	1	0	0	0	0	0	0	0
BDX26	0	D	non-B2	CGA	non-B1	O2b	0	0	0	1	0	0	1	0	0	0	1
BDX27	0	A1	non-B2	non-D	non-B1	ND	0	1	0	1	0	0	1	1	0	0	1
DIJ03	0	B2	II	non-D	non-B1	O2a	0	1	1	1	1	1	1	1	1	1	0
DIJ04	0	C	non-B2	non-D	non-B1	ND	0	1	0	1	0	0	1	0	0	0	1
DIJ06	0	B2	II	non-D	non-B1	O25a	0	1	1	1	1	1	0	0	0	0	0
DIJ07	0	D	non-B2	CGA	non-B1	O17	0	0	0	0	0	0	1	0	0	0	1
DIJ09	0	B2	II	non-D	non-B1	ND	0	1	1	1	0	0	0	0	0	0	1
DIJ10	0	B2	VI	non-D	non-B1	O4	0	1	1	1	1	1	0	1	0	1	0
DIJ11	0	B2	UA	non-D	non-B1	O6	0	1	1	1	1	1	0	1	0	1	1
DIJ12	0	B2	IV	non-D	non-B1	O2b	0	0	0	1	1	1	0	1	0	1	0
DIJ13	0	B2	I	non-D	non-B1	ND	1	1	0	1	0	0	0	0	0	0	0
DIJ14	0	B2	IV	non-D	non-B1	O2b	1	1	1	1	1	1	1	1	0	1	1
DIJ15	0	B2	IV	non-D	non-B1	O2b	1	1	1	1	0	0	0	0	0	0	0
DIJ16	0	B2	I	non-D	non-B1	O6	1	1	1	1	1	1	0	1	1	0	1
DIJ17	0	B2	VI	non-D	non-B1	O4	0	1	1	1	1	1	1	1	1	0	0
DIJ18	0	B1	non-B2	non-D	CC87	ND	0	1	0	1	0	0	1	0	0	0	1
H06	0	B2	II	non-D	non-B1	O6	0	1	1	1	1	1	0	1	1	0	1
H08	0	B2	I	non-D	non-B1	O25b	0	0	0	1	0	0	1	0	0	0	1
H09	0	C	non-B2	non-D	non-B1	ND	0	0	0	1	0	0	0	0	0	0	0
H11	0	F	non-B2	non-D	non-B1	ND	0	1	0	0	0	0	1	0	0	0	1
H12	0	C	non-B2	non-D	non-B1	ND	0	1	0	1	0	0	1	0	0	0	1
H14	0	B1	non-B2	non-D	CC87	ND	0	0	0	1	1	0	1	0	0	0	1
H15	0	B2	II	non-D	non-B1	O6	0	1	1	1	0	0	1	0	0	0	1
H16	0	B2	UA	non-D	non-B1	O75	0	1	1	1	1	1	0	1	0	1	0
H17	0	B2	UA	non-D	non-B1	O16	0	0	0	0	0	0	0	0	0	0	1
H18	0	B2	I	non-D	non-B1	ND	1	1	0	1	0	0	0	0	0	0	1
H19	0	B2	III	non-D	non-B1	O6	0	1	1	1	1	1	0	1	0	1	0
H20	0	B2	VI	non-D	non-B1	O4	0	1	1	1	1	1	0	1	0	1	0
H21	0	B2	I	non-D	non-B1	ND	1	1	0	1	0	0	1	0	0	0	1
H22	0	A	non-B2	non-D	non-B1	ND	0	1	0	0	0	0	1	0	0	0	0
H23	0	B2	UA	non-D	non-B1	O75	0	1	1	0	1	1	0	1	0	1	0
H24	0	D	non-B2	CGA	non-B1	O25b	0	0	0	0	0	0	1	1	1	0	1
H25	0	B2	II	non-D	non-B1	O6	0	1	1	1	1	0	1	1	1	0	1
H27	0	A	non-B2	non-D	non-B1	ND	0	0	0	1	0	0	1	1	0	0	1
H28	0	C	non-B2	non-D	non-B1	ND	0	1	0	1	0	0	1	1	0	0	1
H29	0	C	non-B2	non-D	non-B1	ND	0	0	0	0	0	0	0	0	0	0	1
H30	0	B2	III	non-D	non-B1	O6	0	1	1	1	1	1	0	1	0	1	1
H31	0	B2	I	non-D	non-B1	O25b	0	0	0	1	0	0	1	1	0	0	1
H32	1	C	non-B2	non-D	non-B1	O78	0	1	0	1	0	0	1	1	0	0	1
H33	0	B2	II	non-D	non-B1	O6	0	1	1	1	1	1	1	1	0	0	0
H34	0	B2	II	non-D	non-B1	O6	0	1	1	1	1	1	1	0	0	0	0
H35	0	B2	IV	non-D	non-B1	O2b	1	1	1	1	1	1	0	1	0	1	0
H37	0	A1	non-B2	non-D	non-B1	ND	0	0	0	1	0	0	1	0	0	0	1
LI01	0	B2	UA	non-D	non-B1	O18	0	0	0	1	0	0	0	0	0	0	0

Isolate ID	ESBL phenotype	Phylogenetic group	B2 subgroup	D subgroup	B1 subgroup	Serotype	*neuC*	*iroN*	*sfa*	*fyuA*	*hly*	*cnf1*	*aer*	*papC*	*papGII*	*papGIII*	*traT*
LI02	0	F	non-B2	non-D	non-B1	ND	0	1	0	1	0	0	1	0	0	0	0
LI03	1	B2	I	non-D	non-B1	O25b	0	0	0	1	1	0	1	1	0	0	0
LI04	0	B2	II	non-D	non-B1	O7	1	1	1	1	0	0	0	0	0	0	0
LI05	0	B2	I	non-D	non-B1	O25b	0	1	0	1	0	0	1	1	0	0	1
LI06	0	B2	VI	non-D	non-B1	O4	0	1	1	1	1	1	0	1	0	1	0
LI07	0	B2	UA	non-D	non-B1	ND	0	1	1	1	1	1	1	0	0	0	1
LI08	0	B1	non-B2	non-D	no CC87	ND	0	1	0	0	0	0	1	0	0	0	1
LI09	0	B1	non-B2	non-D	no CC87	ND	0	1	0	0	0	0	1	0	0	0	1
LI10	1	B2	I	non-D	non-B1	O25b	0	0	0	1	0	0	1	1	1	0	1
LI11	0	B2	II	non-D	non-B1	O6	0	1	1	1	0	0	1	0	0	0	1
LI12	0	B2	III	non-D	non-B1	O6	0	1	1	1	1	1	0	1	0	1	0
LM01	0	B2	I	non-D	non-B1	ND	1	1	0	1	1	0	1	0	0	0	1
LM02	0	D	non-B2	CGA	non-B1	O17	0	0	0	1	0	0	0	0	0	0	1
LM03	1	A0	non-B2	non-D	non-B1	ND	0	0	0	0	0	0	1	0	0	0	1
LM08	0	D	non-B2	CGA	non-B1	O25b	0	0	0	1	0	0	0	1	0	0	1
LM09	0	B2	IX	non-D	non-B1	O2a	1	1	0	1	0	0	1	1	1	0	1
LM10	0	D	non-B2	CGA	non-B1	O15	0	0	0	0	0	0	1	1	0	0	1
LM12	0	B2	III	non-D	non-B1	O6	0	1	1	1	1	1	0	1	0	1	0
LM14	0	B2	IV	non-D	non-B1	O2b	1	1	1	1	1	1	0	1	0	1	0
LM17	0	B2	III	non-D	non-B1	O6	0	1	1	1	1	1	0	1	0	1	0
LM19	0	B1	non-B2	non-D	CC87	ND	0	1	0	1	0	0	1	0	0	0	1
LM31	0	B1	non-B2	non-D	CC87	ND	0	1	0	1	0	0	1	0	0	0	1
LM33	1	B2	I	non-D	non-B1	O25b	0	1	0	1	0	0	1	1	0	1	1
LM34	0	B2	I	non-D	non-B1	O25b	0	0	0	1	0	0	1	0	0	0	1
LM36	1	B2	I	non-D	non-B1	O25b	0	1	0	1	0	0	1	1	0	1	1
LM38	0	B2	VI	non-D	non-B1	O4	0	1	0	1	1	1	0	1	0	1	1
LM39	0	B1	non-B2	non-D	CC87	ND	0	1	0	1	0	0	1	0	0	0	1
LM43	0	C	non-B2	non-D	non-B1	ND	0	1	0	1	0	0	1	1	0	0	1
LM45	0	B2	II	non-D	non-B1	O6	0	1	1	1	1	1	1	1	0	0	0
LM49	0	B2	UA	non-D	non-B1	O2b	0	1	1	1	1	1	0	1	0	1	0
LM51	0	B1	non-B2	non-D	CC87	ND	0	0	0	0	0	0	0	0	0	0	1
LM54	0	B2	III	non-D	non-B1	O6	0	1	1	1	1	1	0	1	0	1	0
LM57	0	A	non-B2	non-D	non-B1	ND	0	0	0	0	0	0	0	0	0	0	0
LM58	0	D	non-B2	CGA	non-B1	O7	0	0	0	0	0	0	0	0	0	0	0
LM61	0	B2	I	non-D	non-B1	O16	0	0	0	1	0	0	1	0	0	0	1
LM64	0	B2	UA	non-D	non-B1	ND	0	1	1	1	1	1	0	1	0	1	0
LM67	0	B2	III	non-D	non-B1	O6	0	1	1	1	1	1	0	1	0	1	0
LM68	0	B2	II	non-D	non-B1	O22	0	1	1	1	1	1	0	0	0	0	0
LM69	0	B2	II	non-D	non-B1	O6	0	1	1	1	1	1	1	1	1	0	1
NAN02	1	A1	non-B2	non-D	non-B1	ND	0	1	0	1	0	0	1	1	0	0	1
NAN03	0	B2	IX	non-D	non-B1	O18	1	1	1	1	1	1	1	0	0	0	1
NAN04	0	B2	IV	non-D	non-B1	O2b	1	1	1	1	1	1	0	1	0	1	0
NAN05	1	B1	non-B2	non-D	no CC87	ND	0	1	0	0	0	0	1	0	0	0	1
NAN06	0	A1	non-B2	non-D	non-B1	ND	0	1	0	0	0	0	1	0	0	0	1
NAN08	0	D	non-B2	CGA	non-B1	ND	0	0	0	1	0	0	1	0	0	0	1
NAN09	0	B2	II	non-D	non-B1	O6	0	1	1	1	0	0	0	0	0	0	0
NAN11	0	D	non-B2	no CGA	non-B1	ND	0	0	0	1	0	0	0	1	0	0	0
NAN12	0	B2	IV	non-D	non-B1	ND	1	1	1	1	1	1	0	1	0	1	0

Isolate ID	ESBL phenotype	Phylogenetic group	B2 subgroup	D subgroup	B1 subgroup	Serotype	*neuC*	*iroN*	*sfa*	*fyuA*	*hly*	*cnf1*	*aer*	*papC*	*papGII*	*papGIII*	*traT*
NAN13	0	B2	II	non-D	non-B1	O6	0	1	1	1	1	1	1	0	0	0	0
NAN14	0	B2	VI	non-D	non-B1	ND	0	1	1	1	1	1	0	1	0	1	1
NAN15	0	B2	IV	non-D	non-B1	O2b	0	1	1	1	1	1	0	1	0	1	0
NAN16	0	B2	II	non-D	non-B1	O6	0	1	1	1	1	1	1	0	0	0	0
NAN20	0	B2	II	non-D	non-B1	O6	0	1	1	1	1	1	1	0	0	0	0
NAN21	0	B2	IX	non-D	non-B1	O1	1	1	0	1	0	0	1	1	1	0	1
NAN22	0	B2	IV	non-D	non-B1	O2b	1	1	1	1	0	0	1	0	0	0	0
NAN25	0	B2	IV	non-D	non-B1	O2b	1	1	1	1	1	1	0	1	0	1	0
NAN26	0	B2	IV	non-D	non-B1	O2b	1	1	1	1	1	1	0	1	0	1	0
NAN28	0	B1	non-B2	non-D	no CC87	ND	0	0	0	0	1	1	1	0	0	0	1
NAN29	0	A0	non-B2	non-D	non-B1	ND	0	1	0	0	0	0	0	0	0	0	1
NAN30	0	B2	I	non-D	non-B1	O25b	0	1	0	1	0	0	1	1	0	1	1
NAN31	0	D	non-B2	CGA	non-B1	O17	0	0	0	0	0	0	1	1	0	0	1
NAN32	0	B2	VI	non-D	non-B1	O4	0	1	1	1	1	1	0	1	0	1	0
NAN33	0	A1	non-B2	non-D	non-B1	ND	0	1	0	0	0	0	1	0	0	0	1
NAN34	0	B2	II	non-D	non-B1	O6	0	1	1	1	1	1	0	0	0	0	0
NAN35	0	B2	VI	non-D	non-B1	O4	0	1	1	1	1	1	1	1	0	1	1
NAN36	0	B2	II	non-D	non-B1	O2b	0	1	1	1	0	0	0	0	0	0	0
NAN37	0	D	non-B2	CGA	non-B1	O17	0	0	0	1	0	0	1	1	1	0	1
NAN39	0	B2	UA	non-D	non-B1	O75	0	1	1	1	1	1	0	1	0	1	0
NAN40	0	A1	non-B2	non-D	non-B1	ND	0	0	0	0	0	0	0	0	0	0	0
NAN41	0	B2	VI	non-D	non-B1	O4	0	1	1	1	1	1	0	1	1	1	1
NAN42	0	B1	non-B2	non-D	no CC87	ND	0	0	0	0	0	0	0	0	0	0	1
NAN43	0	D	non-B2	CGA	non-B1	O17	0	1	0	0	0	0	1	0	0	0	1
NAN44	0	B2	UA	non-D	non-B1	O75	0	1	1	1	1	1	0	1	0	1	0
NAN45	0	B2	II	non-D	non-B1	ND	0	1	1	1	1	1	1	0	0	0	0
NAN46	0	B1	non-B2	non-D	CC87	ND	0	1	0	1	0	0	1	0	0	0	1
NAN47	0	B2	II	non-D	non-B1	O6	0	1	1	1	1	1	1	0	0	0	1
NAN50	0	B1	non-B2	non-D	CC87	ND	0	1	0	1	0	0	1	1	0	0	1
NAN51	0	B2	II	non-D	non-B1	O2b	0	1	1	1	1	1	1	1	0	1	0
NIC01	1	B2	IV	non-D	non-B1	O2b	1	1	1	1	1	1	0	0	0	0	1
NIC03	0	B2	IX	non-D	non-B1	O1	1	0	0	1	0	0	0	1	1	0	1
NIC04	0	B2	IX	non-D	non-B1	O18	1	1	1	1	0	0	1	0	0	0	1
NIC05	0	B2	III	non-D	non-B1	O6	0	1	1	1	1	1	0	1	0	0	1
NIC06	0	A1	non-B2	non-D	non-B1	ND	0	1	0	0	0	0	1	1	0	0	1
NIC07	0	A1	non-B2	non-D	non-B1	ND	0	1	0	0	0	0	1	1	0	0	1
NIC08	1	B1	non-B2	non-D	no CC87	ND	0	1	0	0	0	0	1	0	0	0	1
NIC09	0	A1	non-B2	non-D	non-B1	ND	0	0	0	0	0	0	1	1	0	0	1
NIC11	0	B1	non-B2	non-D	no CC87	ND	0	0	0	0	0	0	0	0	0	0	0
NIC12	0	B2	IV	non-D	non-B1	O2b	1	1	1	1	1	1	0	1	0	1	1
PO01	0	B2	UA	non-D	non-B1	O7	1	1	1	1	0	0	0	0	0	0	1
PO02	0	F	non-B2	non-D	non-B1	ND	0	1	0	0	0	0	1	0	0	0	1
PO03	0	B1	non-B2	non-D	no CC87	ND	0	1	0	0	0	0	0	0	0	0	0
PO04	0	B2	II	non-D	non-B1	O25b	0	1	1	1	1	1	0	1	0	1	0
RY02	1	A1	non-B2	non-D	non-B1	O16	0	0	0	0	0	0	0	0	0	0	0
RY03	0	B1	non-B2	non-D	no CC87	ND	0	0	0	0	0	0	0	0	0	0	0
RY05	0	Clade	non-B2	non-D	non-B1	ND	0	0	0	0	0	0	0	0	0	0	0
RY06	0	B2	II	non-D	non-B1	O6	0	1	1	1	1	1	1	0	0	0	0

Isolate ID	ESBL phenotype	Phylogenetic group	B2 subgroup	D subgroup	B1 subgroup	Serotype	*neuC*	*iroN*	*sfa*	*fyuA*	*hly*	*cnf1*	*aer*	*papC*	*papGII*	*papGIII*	*traT*
RY07	0	C	non-B2	non-D	non-B1	ND	0	1	0	0	0	0	1	0	0	0	1
RY08	0	B2	II	non-D	non-B1	O22	0	1	1	1	0	0	0	0	0	0	0
RY09	0	B2	VI	non-D	non-B1	O4	0	1	1	1	1	1	0	1	0	1	0
RY10	0	B2	UA	non-D	non-B1	O2b	1	1	1	1	1	1	0	0	0	0	1
RY11	0	B2	UA	non-D	non-B1	O2b	0	0	0	1	0	0	0	0	0	0	0
RY12	0	D	non-B2	CGA	non-B1	O17	0	1	0	0	0	0	1	0	0	0	1
RY13	0	D	non-B2	no CGA	non-B1	ND	0	0	0	0	0	0	0	0	0	0	0
RY14	0	B2	III	non-D	non-B1	O6	0	1	1	1	1	1	0	1	0	1	0
RY15	0	B1	non-B2	non-D	no CC87	ND	0	1	0	1	0	0	1	0	0	0	1
RY19	0	B2	II	non-D	non-B1	O6	0	1	1	1	1	1	1	0	0	0	0
RY20	0	B2	IX	non-D	non-B1	O45a	1	1	0	1	0	0	1	1	1	0	1
RY21	0	D	non-B2	no CGA	non-B1	ND	0	0	0	0	0	0	0	0	0	0	0
RY22	0	B1	non-B2	non-D	no CC87	ND	0	0	0	0	0	0	0	0	0	0	0
RY24	0	B23	UA	non-D	non-B1	O4	0	1	1	1	1	1	0	1	0	1	1
RY25	0	B2	UA	non-D	non-B1	O2a	0	1	1	1	0	0	0	0	0	0	1
RY26	0	B2	UA	non-D	non-B1	O75	1	0	1	1	1	1	0	0	0	0	1
RY27	0	B2	IV	non-D	non-B1	O2b	1	1	1	1	0	0	1	0	0	0	0
RY28	0	B2	V	non-D	non-B1	O83	1	1	1	1	0	0	1	0	0	0	1
RY29	0	B2	III	non-D	non-B1	O6	0	1	1	1	0	0	0	1	0	1	1
RY33	0	B2	II	non-D	non-B1	O6	0	1	1	1	1	1	1	0	0	0	0
RY34	0	A1	non-B2	non-D	non-B1	ND	0	0	0	0	0	0	0	0	0	0	0
RY3501	0	B2	IV	non-D	non-B1	O2b	1	1	1	1	1	1	0	1	0	1	0
RY3502	0	B2	IV	non-D	non-B1	O2b	1	1	1	1	1	1	0	1	0	1	0
RY36	0	B2	VI	non-D	non-B1	O4	0	1	1	1	1	1	0	1	0	1	0
RY37	0	B2	IV	non-D	non-B1	O2b	1	1	1	1	1	1	0	1	0	1	0
STA1	0	B2	IV	non-D	non-B1	O2b	1	1	1	1	1	1	0	1	0	1	0

*Abbreviations: UA, Unassigned; ND, Not determined; The roman number corresponds to the B2 subgroup. The correspondence with the Achtman MLST schema is as follow: subgroup I = STc131; subgroup II = STc73; subgroup III = STc127; subgroup IV = STc141; subgroup VI = STc12; subgroup VII = STc14; subgroup IX = STc95 [*2*].The most anticipated serotypes in extra-intestinal infections were searched by PCR: O1, O2a, O2b, O4, O6, O7, O12, O15, O16, O17, O18, O22, O25a, O25b, O45a, O75, O78.

References

1. Blanco J, Mora A, Mamani R, López C, Blanco M, Dahbi G, et al. National survey of *Escherichia coli* causing extraintestinal infections reveals the spread of drug-resistant clonal groups O25b:H4-B2-ST131, O15:H1-D-ST393 and CGA-D-ST69 with high virulence gene content in Spain. J Antimicrob Chemother. 2011;66:2011–21. PubMed http://dx.doi.org/10.1093/jac/dkr235

2. Johnson JR, Owens K, Manges AR, Riley LW. Rapid and specific detection of *Escherichia coli* clonal group A by gene-specific PCR. J Clin Microbiol. 2004;42:2618–22. PubMed http://dx.doi.org/10.1128/JCM.42.6.2618-2622.2004

3. Clermont O, Couffignal C, Blanco J, Mentré F, Picard B, Denamur E; COLIVILLE and COLIBAFI groups. Two levels of specialization in bacteraemic Escherichia coli strains revealed by their comparison with commensal strains. Epidemiol Infect. 2017;145:872–82. PubMed http://dx.doi.org/10.1017/S0950268816003010
